# Planar Laser-Based QEPAS Trace Gas Sensor

**DOI:** 10.3390/s16070989

**Published:** 2016-06-28

**Authors:** Yufei Ma, Ying He, Cheng Chen, Xin Yu, Jingbo Zhang, Jiangbo Peng, Rui Sun, Frank K. Tittel

**Affiliations:** 1 National Key Laboratory of Science and Technology on Tunable Laser, Harbin Institute of Technology, Harbin 150001, China; hearkenyi@163.com (Y.H.); chen_cheng0825@163.com (C.C.); yuxin0306@163.com (X.Y.); wozhenkule@sina.com (J.Z.); pengjiangbo_2004@126.com (J.P.); 2 Post-doctoral Mobile Station of Power Engineering and Engineering Thermophysics, Harbin Institute of Technology, Harbin 150001, China; sunsr@hit.edu.cn; 3 Department of Electrical and Computer Engineering, Rice University, 6100 Main Street, Houston, TX 77005, USA

**Keywords:** QEPAS, cylindrical lens, planar laser, H_2_O quantification

## Abstract

A novel quartz enhanced photoacoustic spectroscopy (QEPAS) trace gas detection scheme is reported in this paper. A cylindrical lens was employed for near-infrared laser focusing. The laser beam was shaped as a planar line laser between the gap of the quartz tuning fork (QTF) prongs. Compared with a spherical lens-based QEPAS sensor, the cylindrical lens-based QEPAS sensor has the advantages of easier laser beam alignment and a reduction of stringent stability requirements. Therefore, the reported approach is useful in long-term and continuous sensor operation.

## 1. Introduction

Photoacoustic spectroscopy (PAS) is an effective trace gas sensor technology which is based on the photoacoustic effect. When the laser output is absorbed by a trace gas sample, the absorbed energy is transformed to heat energy by non-radiative processes and will result in an increase of the local temperature and pressure in the sample. Therefore, the absorption of a modulated laser beam in a gas sample leads to the generation of an acoustic wave. The intensity of the acoustic wave is related to the sample concentration which can be detected by a sensitive microphone. However, a microphone-based PAS cell has a low resonance frequency, which makes such a cell more sensitive to environmental and sample gas flow noise. Moreover, the size of a typical photoacoustic cell is relatively large [1].

An improvement of microphone-based PAS is the quartz-enhanced photoacoustic spectroscopy (QEPAS) technique, which was first reported in 2002 [2]. This technique uses a low-cost, commercially available mm-sized piezoelectric quartz tuning fork (QTF) as an acoustic wave transducer which possesses a high detection sensitivity and immunity to ambient acoustic noise [3]. In QEPAS technology, the acoustic energy is accumulated in the sharply resonant QTF and not in a larger photoacoustic cell as in conventional PAS. Therefore, a size limitation of the gas cell no longer exists and the cell volume can be reduced significantly and even the gas cell can be optional depending on the specific application. The total volume of a typical QEPAS acoustic detection module (ADM) is ~4 cm^3^. However, the ADM can be further reduced to ~3 mm^3^, because the volume of the analyzed gas sample is only limited by the dimensions of the QTF. QEPAS has been successfully applied to trace gas detection in numerous applications [4,5,6,7,8,9,10,11], and different sensor architectures leading to specific advantages, such as high sensitivity, selectivity and compactness, were developed. These sensor systems include an off-beam QEPAS sensor [12], an intracavity QEPAS sensor [13], a multi-QEPAS sensor [14], an evanescent wave QEPAS [15,16], an all-fiber QEPAS sensor [17,18], and a scattered light modulation cancellation QEPAS sensor [19]. QEPAS technology was also used for stabilizing the central wavelength of a distributed feedback (DFB) diode laser [20].

QEPAS sensor systems typically employ commercial QTFs with a resonant frequency *f*_0_ of ~32.76 kHz. The length of the QTF prongs is ~3–4 mm and the gap between the two prongs is ~300 µm. The focused laser beam passes through the gap and excites the targeted trace gas species. The position of the laser beam between the prongs should be carefully optimized because the QEPAS signal level is very sensitive to its position [2,18]. The focused laser beam is obtained by using a spherical lens and the diameter of the beam spot at the focal position is of the order of tens of μm [7,8,9,14]. This beam spot should be placed at the optimized position of the QTF to produce the strongest QEPAS signal. This requires precise optical adjustment and the beam propagation must have excellent stability in order to achieve optimum QEPAS sensor performance [18].

In this paper, we report the use of a cylindrical lens to perform laser focusing instead of a spherical lens. The laser beam was shaped as a planar laser and was focused between the gap of the QTF prongs as a line and not as a round beam spot. The length of the line was larger than that of the diameter of the spot. The laser line beam can be adjusted to coincide with the optimized position and the QEPAS signal level was not as sensitive as that for a spot beam. Therefore, the QEPAS system is easier to adjust and reduces the harsh stability requirements. H_2_O was selected as the target analyte to verify the merits of the reported near-infrared cylindrical lens-based QEPAS sensor.

## 2. Experimental Setup

Commercially available QTFs with a resonant frequency *f*_0_ of ~32.76 kHz are usually used in QEPAS sensors. The QEPAS signal amplitude is inversely proportional to the QTF resonant frequency. A QTF with a smaller *f*_0_ will result in a longer effective integration time, which is beneficial in increasing the QEPAS signal. In this paper, a QTF with a *f*_0_ of 30.72 kHz was used as an acoustic wave transducer to improve the sensor sensitivity. The geometries of length, width and thickness of the QTF prongs and the gap between the two prongs are listed in Table 1.

**Figure 1:**
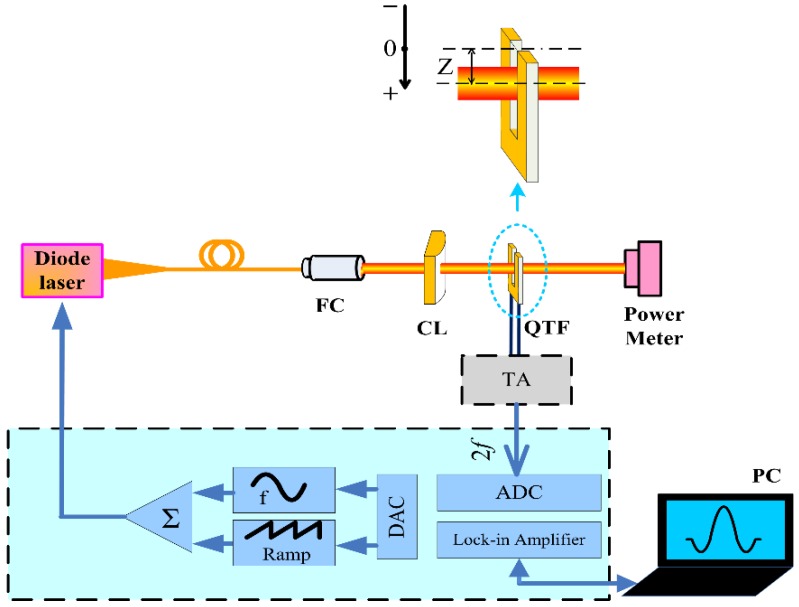
Schematic configuration of the reported QEPAS-based sensor platform.

**Figure 2:**
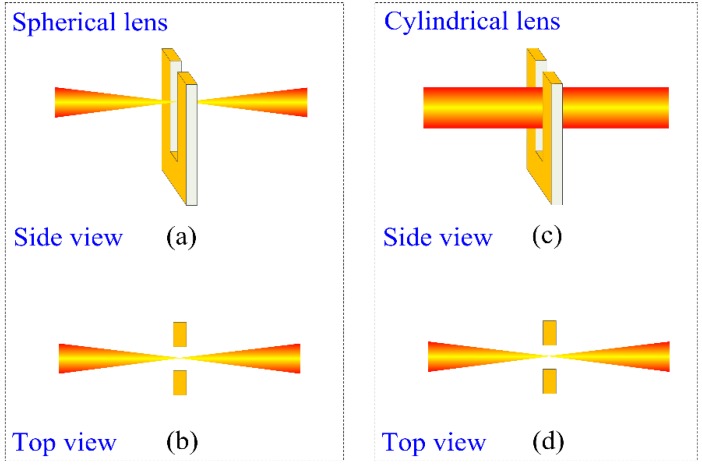
Schematic plot of a QTF and laser beam using different focusing lenses: (**a**) side view for a spherical lens; (**b**) top view for a spherical lens; (**c**) side view for a cylindrical lens; (**d**) top view for a cylindrical lens.

A schematic of the QEPAS-based sensor platform is shown in Figure 1. Wavelength modulation spectroscopy (WMS) with second harmonic detection was utilized for sensitive concentration measurements. Modulation of the laser current was performed by applying a sinusoidal dither to the direct current ramp of the diode laser at half of the QTF resonant frequency (*f* = *f*_0_/2). The piezoelectric signal generated by the QTF is detected by a low-noise trans-impedance amplifier (TA) with a 10 MΩ feedback resistor and converted into a voltage. Subsequently, this voltage was transferred to a lock-in amplifier. A 1.395 µm continuous wave, distributed feedback (CW-DFB) fiber-coupled diode laser with a spectral linewidth of 10 MHz was employed as the laser excitation source. The near-infrared laser beam was collimated using a fiber collimator (FC) with a focal length of 11 mm. Subsequently, for comparison, the laser beam was focused between the QTF prongs using a spherical plano-convex lens (CL) and a cylindrical plano-convex lens, respectively. The schematic plot of the QTF and near-infrared laser beam using different focusing lenses is shown in Figure 2. After passing through the QTF, the laser beam was measured by an optical power meter and used for alignment verification of the QEPAS-based sensor system.

The lens parameters and beam characteristics using two different lenses are shown in Table 2. From Table 2 it is apparent that the length of the laser beam at the focal point using a cylindrical lens is larger than the diameter of the laser spot when a spherical lens was used.

**Figure 3:**
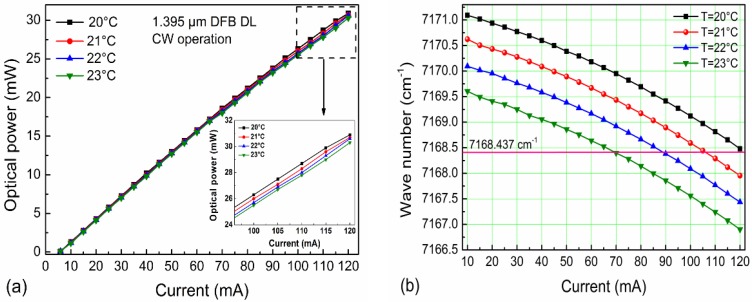
Diode laser output performance at four different temperatures: (**a**) optical power as a function of current; (**b**) diode laser current tuning plots.

The optical power emitted by the near-infrared diode laser operating with a 120 mA drive current was ~30 mW (see Figure 3a). The experimentally determined temperature and current tuning coefficients were −0.51 cm^−1^/°C and −0.0246 cm^−1^/mA, respectively. The DFB diode laser can be current-tuned to target a H_2_O absorption line at 7168.4 cm^−1^ (see Figure 3b), which is free from spectral interference of other molecules. The optimum temperature for the highest optical emitting laser power at the absorption line was 21 °C.

## 3. Results and Discussion

**Figure 4:**
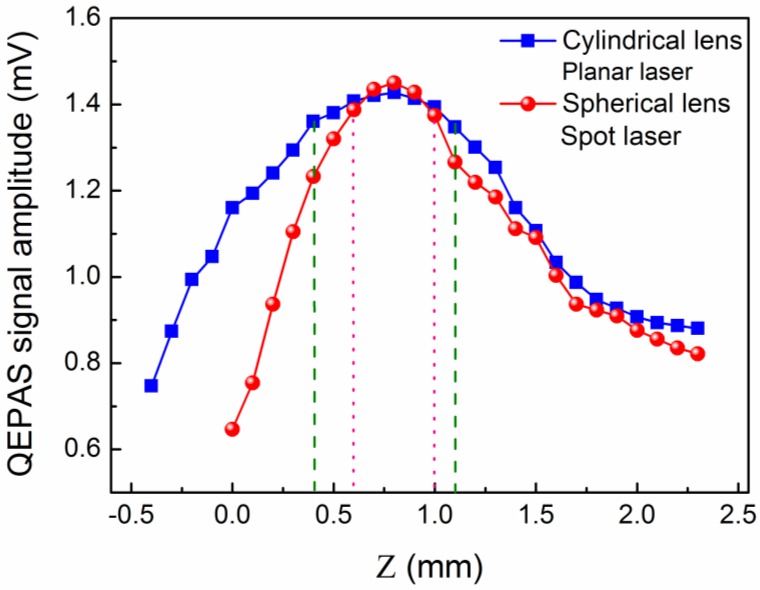
Measured QEPAS signal amplitude as a function of Z (see Figure 1) at a modulation depth of 0.44 cm^−1^ for two different focusing lenses.

Air present in a laboratory environment was employed as the target analyte, which contained 1.09% H_2_O as determined by means of a tunable near-infrared diode laser absorption spectroscopy method. The H_2_O-QEPAS sensor performance using two different plano-convex lenses was evaluated. The impact of the distance (Z, see Figure 1) between the laser beam center and the top of the QTF prongs on the QEPAS signal level was investigated and the experimental results are shown in Figure 4. The modulation depth of the laser wavelength was set to 0.44 cm^−1^. For the cylindrical lens-based QEPAS system, the 2*f* H_2_O-QEPAS signal amplitude increased rapidly with Z when it was <0.4 mm. The peak 2*f* signal amplitude, defined by a signal level decrease to ~95% of the maximum, occurred in the range of Z from 0.4 mm to 1.1 mm (shown as the dashed line in Figure 4). With a further increase of Z, the signal amplitude decreased due to more challenging QTF prong vibrations when the acoustic wave source was at the bottom of the QTF prongs. The negative value of Z means that the laser beam center was higher than at the top of the QTF prongs. The maximum value of Z was set to 2.3 mm, because with a further increase of Z the planar laser beam will be blocked by the QTF. For the spherical lens-based QEPAS system, the 2*f* peak signal amplitude occurred in the range of Z from 0.6 mm to 1 mm (shown as the dotted line in Figure 4). From Figure 4, we can conclude that the maximum signal amplitude for the cylindrical lens-based QEPAS sensor and spherical lens-based QEPAS sensor was almost the same. When compared to the cylindrical lens-based QEPAS sensor system, the signal amplitude of the spherical lens-based QEPAS system changed significantly with Z, especially when Z was <0.8 mm. The ΔZ for the signal peak region of the cylindrical lens-based QEPAS sensor was 0.7 mm (Z from 0.4 mm to 1.1 mm) and that for the spherical lens-based QEPAS sensor was 0.4 mm (Z from 0.6 mm to 1 mm). The value of ΔZ increased by 175%. This means that the cylindrical lens-based QEPAS sensor is insensitive to the position of the QTF prongs. Therefore, the cylindrical lens-based QEPAS sensor is easier to adjust, minimizing the stability requirements, and is advantageous for long-term, continuous operation. In the following reported experiments, an optimum Z of 0.8 mm was chosen to achieve a maximum QEPAS signal amplitude.

**Figure 5:**
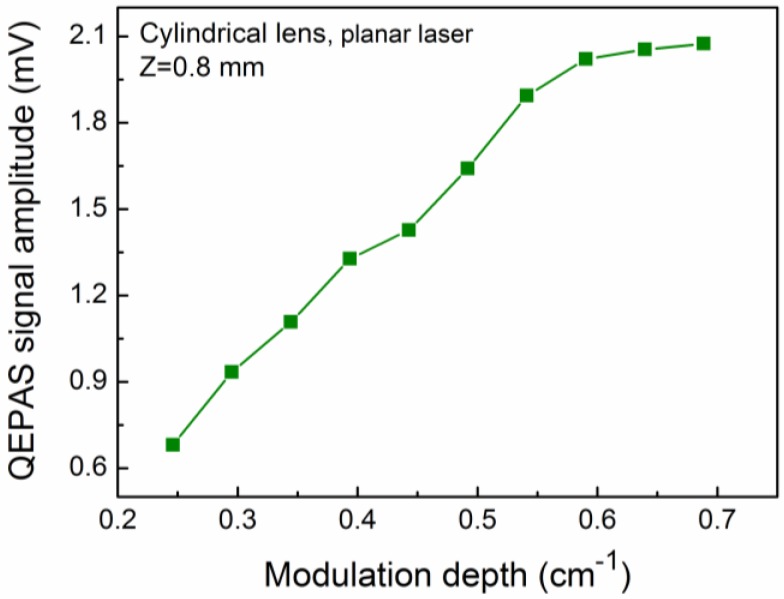
Measured QEPAS signal amplitude as a function of the modulation depth at Z of 0.8 mm for a cylindrical lens-based QEPAS sensor.

The laser wavelength modulation depth was optimized in order to improve the 2*f* QEPAS signal amplitude. The dependence of the cylindrical lens-based QEPAS sensor signal amplitude as a function of the laser wavelength modulation depth is depicted in Figure 5. The QEPAS signal amplitude increased with the modulation depth, but when the modulation depth was >0.59 cm^−1^, no further significant change was observed. Therefore, a modulation depth of 0.59 cm^−1^ was found to be optimum.

**Figure 6:**
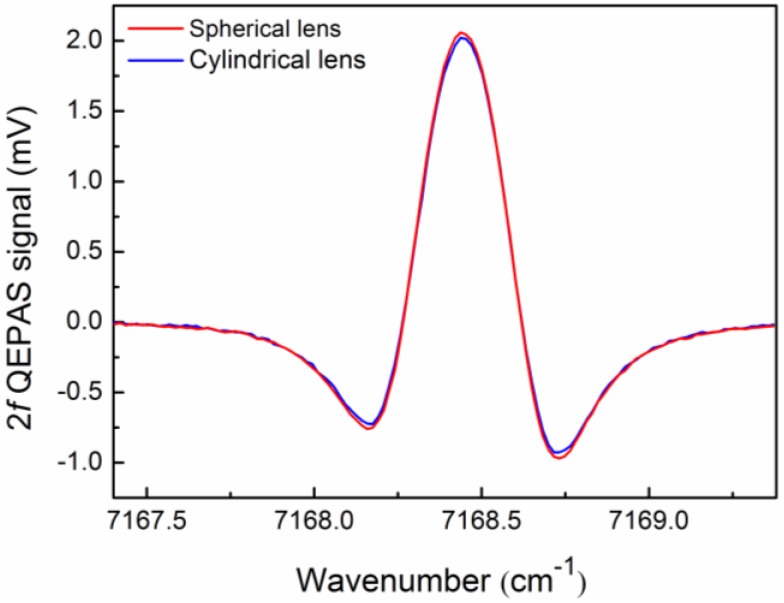
Measured 2*f* QEPAS signal at Z of 0.8 mm and a modulation depth of 0.59 cm^−1^ for cylindrical lens– and spherical lens-based QEPAS sensors, respectively.

The measured 2*f* QEPAS signal at a modulation depth of 0.59 cm^−1^ and a Z value of 0.8 mm for a cylindrical lens-based QEPAS sensor and a spherical lens-based QEPAS sensor is shown in Figure 6, respectively. The signal amplitude was 2.06 mV and 2.02 mV for the spherical lens-based QEPAS sensor and the cylindrical lens-based QEPAS sensor, respectively. The sensor noise was determined as a standard deviation from the signal far from the targeted absorption line. The signal-to-noise ratios (SNRs) calculated from the measured results were 279.5 and 253.7 and this resulted in a minimum detection limit (MDL) for H_2_O of 39 ppm and 42.9 ppm for the spherical lens-based QEPAS sensor and the cylindrical lens-based QEPAS sensor, respectively. There was no obvious difference in the MDLs of the two sensor systems, but the optical adjustment allowance increased dramatically for the cylindrical lens-based QEPAS sensor.

## 4. Conclusions

In conclusion, we demonstrated a novel QEPAS trace gas detection scheme in which a cylindrical lens was used for laser focusing. H_2_O was selected as the target analyte. The laser beam was shaped as planar laser line between the QTF prongs. Compared with the spot beam when a spherical lens was used, the beam line length was much larger than the beam spot diameter. The ΔZ variation represents the optical adjustment when the sensor results in the peak signal level. For a cylindrical lens-based QEPAS sensor, ΔZ was 0.7 mm, and for a spherical lens-based QEPAS sensor, ΔZ was 0.4 mm. We can see that the value of ΔZ was increased by 175% when compared to the spherical lens-based QEPAS sensor. Hence, the cylindrical lens-based QEPAS sensor will be easier to align, reduces the stability requirements and is advantageous for long-term continuous sensor system operation.

## Figures and Tables

**Table 1:** Parameters of custom QTF geometries.

QTF with *f*_0_ (kHz)	Length (mm)	Width (mm)	Thickness (mm)	Gap (mm)
30.72	3.9	0.62	0.36	0.32

**Table 2:** Lens parameters and beam characteristics.

Parameters	Spherical Lens	Cylindrical Lens
Material	CaF_2_	Fused silica
Focal length (mm)	60	75
Transmissivity @ 1395 nm	97%	93%
Beam size at focal point (mm)	Diameter: 0.045	Length: 3.2
		Width: 0.056
